# Reduced colonic mucin degradation in breastfed infants colonized by *Bifidobacterium longum* subsp*. infantis* EVC001

**DOI:** 10.1002/2211-5463.12516

**Published:** 2018-09-17

**Authors:** Sercan Karav, Giorgio Casaburi, Steven A. Frese

**Affiliations:** ^1^ Department of Molecular Biology and Genetics Çanakkale Onsekiz Mart University Turkey; ^2^ Evolve Biosystems, Inc. Davis CA USA; ^3^ Department of Food Science and Technology University of Nebraska Lincoln NE USA

**Keywords:** *Bacteroides*, *Bifidobacterium infantis*, glycome, mucin, mucinlike glycans

## Abstract

Mucin glycoproteins play an important role in protecting the gut epithelium by keeping gut microbes from direct contact with the gut epithelium while allowing for diffusion of small molecules from the lumen to the epithelium. The mucin glycocalyx can be degraded by gut bacteria such as *Bacteroides* and *Akkermansia*, but other bacteria, such as *Bifidobacterium longum* subsp*. Infantis*, cannot consume mucin glycans. Untargeted mass spectrometry profiles were compared to microbiome profiles to assess how different gut microbiomes affect colonic mucin degradation. Samples obtained from nine infants colonized by *Bifidobacterium infantis* EVC001 and from 10 infants colonized by higher levels of mucolytic taxa (controls), including *Bacteroides*, were compared. Previously performed untargeted nano‐high‐performance liquid chromatography‐chip/time‐of‐flight mass spectrometry was used to detect and quantify glycans originating from colonic mucin. Colonic mucin‐derived *O*‐glycans from control infants composed 37.68% (± 3.14% SD) of the total glycan structure pool, whereas colonic mucin‐derived *O*‐glycans made up of only 1.78% (± 0.038% SD) of the total in *B. infantis* EVC001 samples. The relative abundance of these colonic mucin‐derived *O*‐glycans in the total glycan pool was higher among control, 26.98% (± 8.48% SD), relative to *B. infantis‐*colonized infants, 1.68% (± 1.12% SD). Key taxa, such as *Bacteroidaceae*, were significantly and positively correlated with the abundance of these structures, while *Bifidobacteriaceae* were significantly and negatively associated with these structures. These results suggest that colonization of infants by *B. infantis* may diminish colonic glycan degradation and help maintain barrier function in the gastrointestinal tract of infants.

**Abbreviations**
ACN: acetonitrile
FA: formic acid
FDR: false discovery rate
GH: glycosyl hydrolase
HMO: human milk oligosaccharide
HPLC: high‐performance liquid chromatography
OS: oligosaccharide
OTU: operational taxonomic unit
PCoA: principal coordinate analysis
TOF: time of flight

Mucins are highly glycosylated *O*‐linked glycoproteins secreted by exocrine glands and mucosa. They contain a wide variety of glycan structures, which make up 80% of the glycoprotein mass. These glycans attach to the polypeptide backbone via serine or threonine residues with *N*‐acetylgalactosamine. Eight *O*‐glycan cores have been identified 1. Among these cores, core 1‐, 2‐, 3‐, and 4‐derived glycans are predominantly found in intestinal mucins. Specifically, core 1 (Galβ1–3GalNAcα1‐Ser/Thr) and core 2 (Galβ1,3(GlcNAcβ1,6)GalNAcα1‐Ser/Thr) structures are found in gastric and duodenal mucins, whereas colonic mucins contain predominantly core 3, 4 (GlcNAcβ1,6 (GlcNAcβ1,3) GalNAcαSer/Thr) elongated structures 2, 3. Mucin degradation by mucolytic taxa like *Bacteroides* is achieved with a wide variety of enzymes, such as proteases, sulfatases, fucosidases, neuraminidases, β‐galactosidases, α‐*N*‐acetylgalactosaminidases, α‐*N*‐acetylglucosaminidases, and exo/endo‐β‐*N*‐acetyl‐glucosaminidases 4. Although the presence of mucin‐consuming taxa is associated with increased mucin production, it is unclear whether this is a beneficial trait as the breakdown of the mucin barrier is often associated with negative impacts to the underlying gut epithelium 5, and its loss can be a pathway to infection 6, loss of gut epithelial barrier function 7, or spontaneous inflammation resembling colitis 8. Further, mucin degradation provides a niche that fosters taxa whose cytotoxic products can lead to colon cancer 9.

Human milk contains structurally analogous carbohydrates in the form of *N*‐linked and *O*‐linked glycoproteins and glycolipids 10 as well as free glycans, known as human milk oligosaccharides (HMOs) 11. Several gut bacteria found in breastfed infants are able to release and consume mucin glycans (e.g., *Bacteroides*), and while some species of *Bifidobacterium* (e.g., *B. bifidum*) are able to grow on mucin as a sole carbon source, others can not (e.g., *Bifidobacterium infantis*) 12. Interestingly, the taxa that consume both mucin glycans and HMOs appear to use the same glycolytic capacities and regulatory networks to consume these structures, whereas specialized taxa consume HMOs in unique pathways, and appear to only express these genes in response to a limited number of carbohydrates 13, 14, 15. Thus, adaptation to these two structurally similar carbon sources appears to be highly specific among different infant gut‐associated bifidobacteria. If this is true, then the role of mucolytic taxa in the infant gut is unclear. Further, to what extent is mucus degraded in the infant gut and which taxa are responsible? To begin to address these questions, a library of known colonic mucin‐derived *O*‐glycans was compiled and used to query untargeted mass spectra of fecal samples from infants from a previous study 16, 17. We hypothesized that the previously demonstrated modification of the gut microbiome resulted in the modulation of mucin degradation by gut microbes. A second part of the hypothesis was that colonization with *B. infantis,* which does not degrade mucin, and the subsequent reduction in mucolytic taxa would diminish mucin degradation, as measured by the abundance of freed colonic mucin‐derived *O*‐glycans in the infant's stool.

## Materials and methods

### Fecal sample collection and analysis

To examine the effect of *Bifidobacterium longum* subsp. *infantis* (*B. infantis*) EVC001 colonization on gut mucin degradation at day 29 postnatal, fecal samples were collected from healthy, breastfed infants fed 1.8 × 10^10^ CFU per day *B. longum* subsp. *infantis* EVC001 from Day 7 postnatal to Day 29 postnatal (*n *=* *9) and healthy breastfed infants who were not fed *B. longum* subsp. *infantis* EVC001 (*n *=* *10). These samples were randomly selected from the larger study population for additional analysis using untargeted mass spectrometry data that had been collected initially for the study of human milk glycans found in the infant's feces. The initial clinical study was a partially randomized study, and subject populations, as well as ethical approval for sample collection, were previously noted 16. Demographics describing the randomly selected subset of the total population examined in this analysis is presented in Table S1.

The microbiome composition of these samples was also previously determined. These data are publicly deposited in the NCBI SRA (PRJNA390646) and were analyzed using QIIME 1.9.1 18 as previously described 17. Briefly, paired‐end sequencing of the V4 region of the 16S rRNA gene was performed on an Illumina MiSeq at the University of California Davis Genome Center (Davis, CA). Open‐reference operational taxonomic unit (OTU) picking was completed using UCLUST at 97% identity, and low‐abundance OTUs were removed as recommended 19. Across the full data set, there was a mean of 9216 reads (SD ± 4505 reads) per sample and samples with at least than 2779 reads were included for analysis. Here, 19 samples (*n* = 9, 10) matching paired mass spectrometry data were selected from this overall population and analyzed in the context of the untargeted spectra as described below.

### Analysis of spectra obtained with Nano‐high‐performance liquid chromatography‐chip/time‐of‐flight mass spectrometry (nano‐LC‐MS)

Oligosaccharide (OS) isolation and purification from these fecal samples were performed previously and reported by Frese *et al*. 17. The structures of human colonic mucin *O*‐glycans were characterized by analysis on a nano‐HPLC‐Chip‐TOF mass spectrometer using the methods described by Davis *et al*. 20, and this approach was previously reported for these samples where the concentration of structurally similar HMOs was determined 17. Briefly, the high‐performance liquid chromatography (HPLC) system used was an Agilent 1200 series unit with a microfluidic chip, which was coupled to an Agilent 6220 series time of flight (TOF) mass spectrometer via chip cube interface. The capillary pump on the chromatography unit loaded the sample onto the 40‐nL enrichment column at a flow rate of 4.0 μL·min^−1^ with a 1 μL injection volume. A nano pump was used for analyte separation on the analytical column, which was 75 × 43 mm and packed with porous graphitized carbon. Separation was accomplished using a binary gradient of aqueous solvent A [3% acetonitrile (ACN)/water (v/v) in 0.1% formic acid (FA)] and organic solvent B [90% ACN/water (v/v) in 0.1% FA] using a method developed for HMO separation (7, 8). The sample was introduced into the TOF mass spectrometer via electrospray ionization, which was tuned and calibrated using a dual nebulizer electrospray source with calibrant ions ranging from m/z 118.086 to 2721.895, and data were collected in the positive mode. These untargeted spectra were analyzed in the present study as described below.

### Glycan data analysis

The untargeted mass spectra were collected (as above) and analyzed using Agilent MassHunter Workstation Data Acquisition version B.02.01 on the nano‐HPLC‐Chip‐TOF. The ‘Find Compounds by Molecular Feature’ function of the software was used to identify mucin glycan species within 20 ppm of theoretical masses. Compound abundances were expressed as volume in ion counts that corresponded to absolute abundances of the compounds in each sample. 1HexNAc‐1NeuAc, 1HexNAc‐1Hex‐NeuAc, 2HexNAc‐1NeuAc, 2HexNAc‐1Hex‐1Fuc, 2HexNAc‐1Hex‐1NeuAc, 2HexNAc‐1Hex‐2Fuc, 3HexNAc‐1Hex‐1Fuc, 2HexNAc‐1Hex‐1Fuc‐1NeuAc, 2HexNAc‐1Hex‐1Fuc‐2NeuAc, 3HexNAc‐1Hex‐2NeuAc and 3HexNAc‐1Hex‐2Fuc‐1NeuAc were monitored as they are discriminatively human colonic mucin *O*‐glycans 21.

### Statistical analysis

Multiple *t*‐tests, corrected using the Holm–Sidak method for multiple comparisons, were carried out in Graph Pad Prism 7 (graphpad Software, La Jolla, CA, USA). Wilcoxon rank‐sum test was used for single comparisons. *P* values, or adjusted *P* values, of 0.05 or less in comparisons were considered significantly different. Differences in bacterial community composition and colonic mucin‐derived *O*‐glycans were calculated in several, complementary ways. First, a weighted UniFrac distance matrix 22 was used to visualize differences in community composition according to treatment group using principal coordinate analysis (PCoA). Second, a Bray–Curtis dissimilarity index between all colonic mucin‐derived *O*‐glycan species was visualized via PCoA. To evaluate the effect size of EVC001 colonization, both weighted UniFrac and Bray–Curtis dissimilarity matrices were tested via Permanova multivariate comparisons with 999 permutations and FDR‐corrected *P*‐values. Mantel tests were used to assess significant relationships between the phylogenetic distance of the bacterial communities and the colonic mucin‐derived *O*‐glycan abundance. Colonic mucin‐derived *O*‐glycan abundance was transformed to dissimilarity matrices using Euclidean distance, while phylogenetic distance was obtained via the weighted UniFrac algorithm. Tests were performed using Pearson's product‐moment correlation coefficient (*r*) with 999 permutations and a two‐tailed test.

To compute specific correlations between observations of bacterial taxa and colonic mucin‐derived *O*‐glycan structures, a Spearman's ρ test was used. Raw correlation statistics were tested for likelihood using Fisher's *Z* transformation and *P*‐values corrected via Benjamini–Hochberg false discovery rate (FDR) procedure.

## Results and Discussion

### Comparisons of the gut microbiome among a subselected cohort

**Figure 1:**
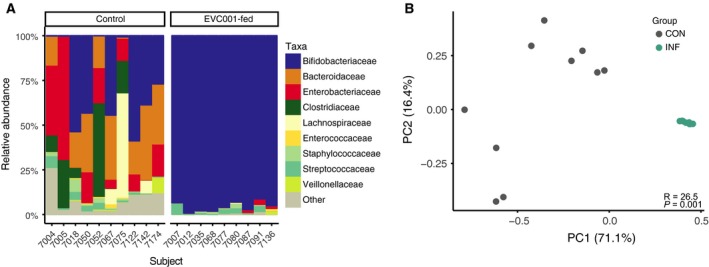
(A) The relative abundance of the taxa, as reported at the family level by Frese *et al*. 2017, of each fecal sample in this analysis. (B) PCoA of the gut microbiome at the family level; control (CON) samples are shown as gray points, and EVC001‐fed infant samples are shown as teal points. 87.5% of total variation was described in the first two principal components (PC1 and PC2). PERMANOVA comparisons identified a significant difference between the two treatment groups by composition (*R *=* *26.5, *P *=* *0.001).

The gut microbiome of samples analyzed by mass spectrometry from infants profiled by Frese *et al*. is shown in Fig. 1A. Infants were fed 1.8 x 10^10^ CFU per day of *B. infantis* EVC001 for 21 days from Day 7 to Day 29 postnatal. Infants fed EVC001 had significantly high levels of *Bifidobacterium* and significantly lower levels of *Bacteroides* than control infants not fed *B. infantis* EVC001. *Bacteroides* was the predominant mucolytic taxon identified in the samples 23. Notably, *B. infantis* fails to grow on colonic mucin as a sole carbon source 15, and the relative abundance of *Bacteroidaceae* was negatively correlated with the abundance of *Bifidobacteriaceae* (Spearman's ρ = −0.65; *P *=* *0.0029). Among the 19 samples profiled by nano‐HPLC‐Chip‐TOF here, the gut microbiome profiles for nine infants fed *B. infantis* EVC001 were significantly different as tested by PERMANOVA (*R *=* *26.5, *P* = 0.001) from that of the 10 control infants (Fig. 1B). Even when all *Bifidobacterium‐*matched reads were filtered from the samples, the residual microbial communities were still significantly different according to treatment group when compared by PERMANOVA (*R *=* *13.8, *P* = 0.001).

### Fecal glycomics indicates differences among release of colonic mucin‐derived *O*‐glycans from glycoproteins *in vivo*

**Figure 2:**
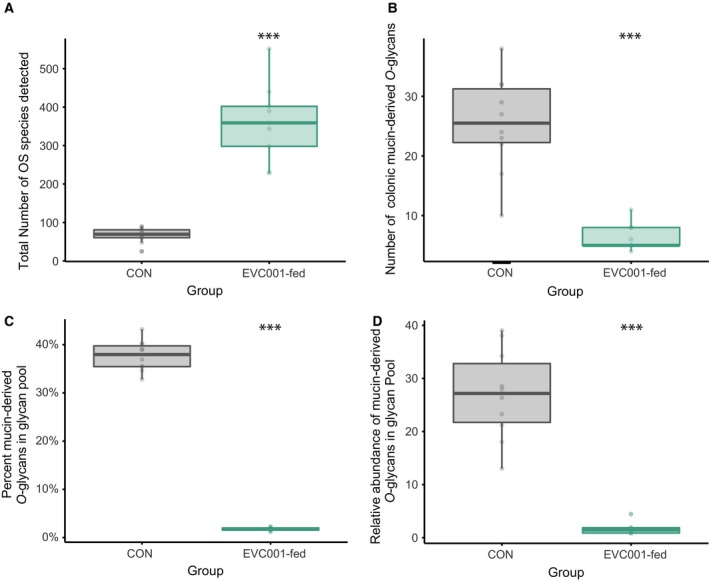
Comparison of fecal glycome and colonic mucin‐derived *O‐*glycans of control and EVC001‐fed infant feces. (A) Total number of OS detected across treatment groups. (B) Number of colonic mucin‐derived *O‐*glycans across treatment groups. (C) Relative abundance of the total number of colonic mucin‐derived *O‐*glycans in the total OS pool across treatment groups. (D) Percent of the OS assigned to colonic mucin‐derived *O‐*glycans in the total OS abundance across treatment groups.

The total OS compositions of the samples were determined by the untargeted approach of nano‐HPLC‐Chip‐TOF. The compounds were characterized using previously published libraries 21, 24, 25, 26. Among these compositions, free HMOs and free colonic mucin‐derived *O*‐glycans were found in the infant fecal glycome. The main focus of this study was to understand the degradation of human colonic mucin glycans by different gut microbiome profiles. This was determined as the difference between a gut microbiome from infants colonized with *B. infantis* EVC001 and microbiomes with a greater abundance of mucolytic taxa, such as *Bacteroidaceae*. As target molecules, 1HexNAc‐1NeuAc, 1HexNAc‐1Hex‐NeuAc, 2HexNAc‐1NeuAc, 2HexNAc‐1Hex‐1Fuc, 2HexNAc‐1Hex‐1NeuAc, 2HexNAc‐1Hex‐2Fuc, 3HexNAc‐1Hex‐1Fuc, 2HexNAc‐1Hex‐1Fuc‐1NeuAc, 2HexNAc‐1Hex‐1Fuc‐2NeuAc, 3HexNAc‐1Hex‐2NeuAc and 3HexNAc‐1Hex‐2Fuc‐1NeuAc were selected as typical human colonic mucin glycans, as shown by Robbe *et al*. 21. The mass spectrometry monitoring these structures showed that the number of total OS structures (including isomers and anomers) in samples from control and EVC001‐fed infants ranged from 67.4 ± 19.81 and 360.44 ± 102.52, respectively (*P *<* *0.001; Fig. 2A). Although the control samples contained fewer total OS structures, the number of freed human colonic mucin‐derived *O*‐glycans of the total OS was significantly higher 25.4 (±8.09), whereas only 6.33 (±2.24) structures were colonic mucin‐derived *O*‐glycans in samples from EVC001‐fed infants (*P *<* *0.001, Wilcoxon test; Fig. 2B). As a proportion, the relative abundance of colonic mucin‐derived *O*‐glycans was significantly higher in control samples than in samples from EVC001‐fed infants in terms of both the number of structures (37.68% ± 3.14% and 1.78% ± 0.385%, respectively; Fig. 2C, *P* < 0.001, Wilcoxon test) and their proportion of the total OS profile (26.98% ± 8.48% and 1.68 ± 1.12%, respectively; Fig. 2D, *P* < 0.001, Wilcoxon test).

### Associations between colonic mucin degradation and the fecal microbiome

**Figure 3:**
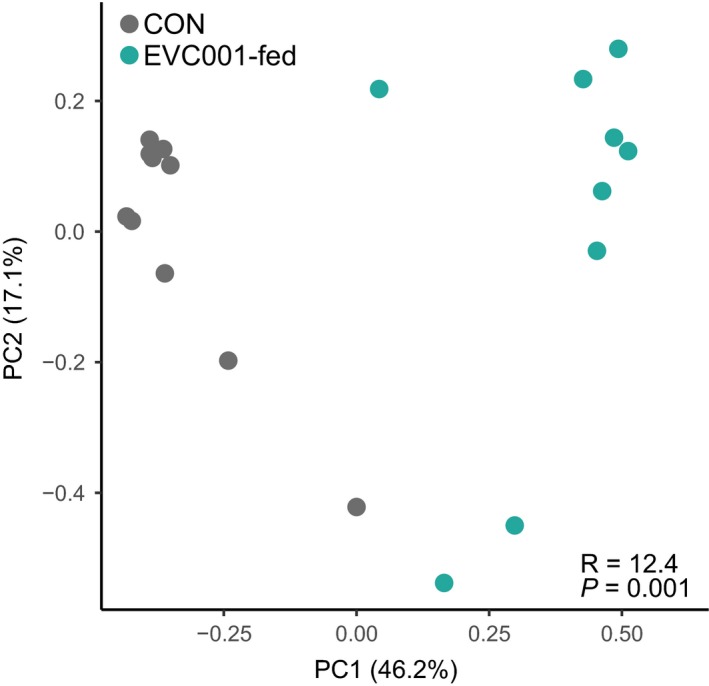
PCoA of colonic mucin‐derived *O‐*glycan composition among samples using a Bray–Curtis dissimilarity index; control (Con) samples are shown as gray points, and EVC001‐fed infant samples are shown as teal points. 63.3% of total variation was explained in the first two principal components (PC1 and PC2). PERMANOVA comparisons identified a significant difference between the two groups, with respect to colonic mucin‐derived *O‐*glycan composition (*R* = 12.4; *P *=* *0.001).

Overall, freed colonic mucin *O*‐glycan composition differed significantly between EVC001‐colonized and control infants when tested via PERMANOVA (*R* = 12.4, *P *=* *0.001; Fig. 3). However, to compare the composition of the gut microbiome with the abundance of both total colonic mucin‐derived *O*‐glycans and the specific structures monitored here, a Mantel test was used to correlate these structures with the overall microbiome composition. Broadly, the total colonic mucin‐derived *O*‐glycan abundance was significantly correlated with the microbiome composition (Mantel's *R* = 0.39, *P* = 0.01). Of these, only 1_0_0_1 (Mantel's *R* = 0.22, *P* = 0.05), 1_1_0_1 (Mantel's *R* = 0.3, *P* = 0.027), 2_1_1_0 (Mantel's *R* = 0.46, *P* = 0.003), 2_1_1_1 (Mantel's *R* = 0.42, *P* = 0.003), 2_1_2_0 (Mantel's *R* = 0.69, *P* = 0.001), 3_1_1_0 (Mantel's *R* = 0.35, *P* = 0.011), and 3_1_2_1 (Mantel's *R* = 0.37, *P* = 0.005) were significantly associated with specific microbiome compositions.

To examine the interactions of the gut microbiome and the colonic mucin‐derived *O*‐glycan species, a Pearson correlation was calculated for all taxa and structures in the samples, as well as the total abundance and proportion of colonic mucin‐derived *O*‐glycan species (Table 1). *Bifidobacteriaceae* abundance was significantly and negatively correlated with the abundance of colonic mucin‐derived *O‐*glycans, as a whole (Spearman's ρ = −0.66, *P* = 0.04), whereas *Bacteroidaceae* was significantly and positively correlated with the abundance of 1_0_0_1, 2_1_1_1, 3_1_2_1, and 2_1_2_0 (Table 2). Interestingly, *Enterobacteriaceae,* who are unlikely to degrade mucus themselves, were significantly correlated with the overall abundance of colonic mucin‐derived *O*‐glycans (Spearman's ρ = 0.061, *P* = 0.04) as well as 1_0_0_1 (Spearman's ρ = 0.63, *P* = 0.03), and 2_1_2_0 (Spearman's ρ = 0.71, *P* = 0.003). Similarly, *Clostridiaceae* (Spearman's ρ = 0.7, *P* = 0.01) and *Planococcaceae* (Spearman's ρ = 0.63, *P* = 0.03) were significantly correlated with 2_1_1_0. While *Pasteurellaceae* were significantly correlated with the overall abundance of colonic mucin‐derived *O*‐glycans, they were not associated with specific *O‐*glycan species. Interestingly, three of these colonic mucin‐derived *O*‐glycan species (1_1_1_0, 3_1_1_0 and 3_1_2_1) were significantly associated with microbiome compositions, but no individual taxa were responsible for this association.

**Table 1:** Colonic mucin‐derived *O‐*glycan structure, composition, mass, and volume in samples from the two treatment groups

Glycan code	Composition	Neutral mass	Log_10_ volume control [Mean (±SD)]	Log_10_ volume EVC001‐fed [Mean (±SD)]	Holm–Sidak adjusted *P* value
1_0_0_1	1HexNAc‐1NeuAc	512	7.19 (7.08)	5.44 (5.55)	0.010659
1_1_0_1	1HexNAc‐1Hex‐1NeuAc	675	5.96 (6.01)	4.06 (4.53)	0.09968
2_0_0_1	2HexNAc‐1NeuAc	716	5.93 (6.16)	0 (0)	0.258702
2_1_1_0	2HexNAc‐1Hex‐1Fuc	735	6.17 (6.23)	0 (0)	0.09968
2_1_0_1	2HexNAc‐1Hex‐1NeuAc	878	5.9 (6.11)	5.5 (5.56)	0.502866
2_1_2_0	2HexNAc‐1Hex‐2Fuc	879	6.45 (5.95)	5.46 (5.51)	3.13E‐06
3_1_1_0	3HexNAc‐1Hex‐1Fuc	936	6.43 (6.31)	5.32 (5.43)	0.015609
2_1_1_1	2HexNAc‐1Hex‐1Fuc‐1NeuAc	1024	7.31 (7.01)	5.42 (5.54)	0.000189
2_1_1_2	2HexNAc‐1Hex‐1Fuc‐2NeuAc	1315	7.08 (7.26)	6.71 (7.02)	0.502866
3_1_0_2	3HexNAc‐1Hex‐2NeuAc	1372	6.43 (6.52)	5.25 (5.4)	0.141386
3_1_2_1	3HexNAc‐1Hex‐2Fuc‐1NeuAc	1373	7.52 (7.28)	5.77 (5.85)	0.000895

**Table 2:** Correlational analysis comparing microbiome composition (Mantel test) and specific bacterial families (Spearman's ρ) with colonic mucin‐derived *O‐*glycans

Mucin‐glycan Structures	Mantel *r*	Mantel *P*‐value	Bacterial family	Spearman's ρ	*P*‐value (FDR)
Total	0.39	0.01	*Bifidobacteriaceae*	−0.66	0.04
			*Enterobacteriaceae*	0.63	0.04
			*Pasteurellaceae*	0.61	0.04
1_0_0_1	0.22	0.05	*Bifidobacteriaceae*	−0.65	0.03
			*Enterobacteriaceae*	0.63	0.03
			*Bacteroidaceae*	0.63	0.03
1_1_0_1	0.3	0.027			
2_0_0_1	0.03	0.826			
2_1_0_1	0.07	0.590			
2_1_1_0	0.46	0.003	*Clostridiaceae*	0.7	0.01
			*Planococcaceae*	0.63	0.03
			*Bifidobacteriaceae*	−0.63	0.03
2_1_1_1	0.42	0.003	*Bacteroidaceae*	0.69	0.01
			*Bifidobacteriaceae*	−0.67	0.01
2_1_1_2	0.19	0.109			
2_1_2_0	0.69	0.001	*Bifidobacteriaceae*	−0.82	7.85E‐05
			*Bacteroidaceae*	0.72	0.003
			*Enterobacteriaceae*	0.71	0.003
3_1_0_2	0.25	0.062			
3_1_1_0	0.35	0.011			
3_1_2_1	0.37	0.005			

The broad associations between diminished abundance, number, and proportion of colonic mucin‐derived *O*‐glycans and colonization by *B. infantis* EVC001 was reflected in the negative correlations between the abundance of *Bifidobacteriaceae* and both total colonic mucin‐derived *O*‐glycan abundance and four of the mucin‐derived *O‐*glycan species monitored here. Similarly, the abundance of a known mucin‐degrading family, *Bacteroidaceae*, was positively correlated with the abundance of these same structures. Many *Bacteroides* species allocate a large proportion of their genome to harvesting polysaccharides, including mucin 27, and the significant positive correlation with colonic mucin‐derived *O*‐glycan concentrations supports these previous findings, even though 16S rRNA gene sequencing here does not confidently distinguish between OTUs at the species level. Many of the genes associated with polysaccharide utilization common to mucin‐degrading Bacteroides are highly active on mucin glycoproteins, including the *O*‐glycan cores found in human colonic mucin, as evidenced here. *Bacteroides* can grow on mucin a sole carbon source and have specific transcriptional responses to incubation with mucin 13. In particular, *Bacteroides* broadly possess enzymes from glycosyl hydrolase (GH) family GH 84, GH 85, GH 89, GH 101, and GH 129 that are active on mucin glycoproteins and may facilitate the release of these glycans from the mucin protein 13, 27, 28. The release of these structures may facilitate the growth of taxa which are potentially pathogenic (e.g., *Clostridiaceae* and *Enterobacteriaceae*), and sialylated and fucosylated glycans derived from host mucin have been shown to play a major role in disease pathogenesis by *Salmonella*,* Clostridium difficile,* and cytotoxic *Escherichia coli* 9 and taxa that act as reservoirs of virulence factors in the infant gut 29.

## Conclusion

The human gastrointestinal epithelium is protected by a layer of mucus. Mucin, as a glycoprotein, is coated with a wide variety of conjugated glycans that can serve as a carbon source for mucolytic bacteria, and their release may facilitate the growth of other taxa such as *Enterobacteriaceae* and *Clostridiaceae*. Specifically, human colon mucins contain unique *O*‐glycan structures that can be utilized only by few mucolytic taxa, and in breastfed infants, the predominant mucolytic taxon was *Bacteroidaceae*. These bacteria harbor multiple GH‐encoding genes that facilitate the liberation of colonic mucin *O*‐glycan from glycoproteins. Our results show that gut microbiomes of infants colonized by *B. infantis* have diminished mucin degradation, as evidenced by reduced abundance and diversity of freed colonic mucin‐derived *O‐*glycans, as well as the negative correlations between these and *Bifidobacteriaceae*. *B. infantis* is not able to cleave colonic mucin‐derived *O*‐glycans, and their colonization is associated with diminished populations of *Bacteroidaceae* 17. Infants with a gut microbiome with higher relative abundance of mucolytic taxa have enhanced mucin degradation, and this may have long‐term health consequences, or increase their susceptibility to infection from taxa harboring virulence factors via cross‐feeding 6, 9, 29. Further studies are required to determine the specific negative effects of mucin degradation, the role of these taxa in diminishing host gut barrier function, and any functional consequences to the host.

## Author contributions

SK performed the analysis of the mass spectra. SF and GC performed statistical tests and microbiome analyses. All authors contributed to the study, read, and approved the final manuscript.

## Conflict of interest

SAF and GC are employed by Evolve Biosystems, Inc., which funded the study from which these samples were derived.

## Supporting information


**Table S1.** Study participant demographics showing the mean (±standard deviation) or number per group.
