# Supplementary material for: Reduced colonic mucin degradation in breastfed infants colonized by Bifidobacterium longum subsp. infantis EVC001
Source: FEBS Open Bio. 2018 Sep 17;8(10):1649–57. doi: 10.1002/2211-5463.12516 (PMC6168692; doi:10.1002/2211-5463.12516)
Supplement: Supplementary file 1 — Table S1. Study participant demographics showing the mean (±standard deviation) or number per group. [file FEB4-8-1649-s001.docx]

Table 1. Study participant demographics showing the mean (+/- standard deviation) or number per group.

| Measure | Controls | EVC001-colonized | FDR-adjusted  P value | Fisher's Exact Test |
| --- | --- | --- | --- | --- |
| Number of samples | 10 | 9 |  |  |
| Number of CS births | 3 | 1 |  | 0.582 |
| Hours in labor | 24.2 (26.22) | 7 (5.32) | 0.17 |  |
| Received antibiotics for Labor | 4 | 3 |  | 1.00 |
| Labor Complications occurred | 4 | 3 |  | 1.00 |
| Calculated Gestational Age | 40.4 (0.83) | 38.98 (1.24) | 0.06 |  |
| Number of Males | 5 | 4 |  | 1.00 |
| Birth weight (g) | 3730.79 (717.59) | 3269.96 (331.12) | 0.17 |  |
| Birth length (cm) | 51.13 (2.2) | 49.18 (1.83) | 0.17 |  |
| Baby received antibiotics | 1 | 0 |  | 1.00 |
| Complications at birth | 2 | 0 |  | 0.47 |
| Infant Formula consumed before discharge | 1 | 0 |  | 1.00 |
| Maternal Pre-pregnancy BMI | 25.41 (3.07) | 23.51 (2.84) | 0.26 |  |
| Maternal Pregnancy weight gain (kg) | 14.06 (5.48) | 14.32 (3.63) | 0.91 |  |
| GBS positive mothers | 4 | 4 |  | 1.00 |
| Primiparous mother | 9 | 2 |  | 0.01 |
| Maternal age | 31.2 (4.66) | 33.11 (3.76) | 0.40 |  |
